# iRefIndex: A consolidated protein interaction database with provenance

**DOI:** 10.1186/1471-2105-9-405

**Published:** 2008-09-30

**Authors:** Sabry Razick, George Magklaras, Ian M Donaldson

**Affiliations:** 1 The Biotechnology Centre of Oslo, University of Oslo, P.O. Box 1125 Blindern, 0317 Oslo, Norway; 2 Biomedical Research Group, Department of Informatics, University of Oslo, P.O. Box 1080 Blindern, 0316 Oslo, Norway; 3 Department for Molecular Biosciences, University of Oslo, P.O. Box 1041 Blindern, 0316 Oslo, Norway

## Abstract

**Background:**

Interaction data for a given protein may be spread across multiple databases. We set out to create a unifying index that would facilitate searching for these data and that would group together redundant interaction data while recording the methods used to perform this grouping.

**Results:**

We present a method to generate a key for a protein interaction record and a key for each participant protein. These keys may be generated by anyone using only the primary sequence of the proteins, their taxonomy identifiers and the Secure Hash Algorithm. Two interaction records will have identical keys if they refer to the same set of identical protein sequences and taxonomy identifiers. We define records with identical keys as a redundant group. Our method required that we map protein database references found in interaction records to current protein sequence records. Operations performed during this mapping are described by a mapping score that may provide valuable feedback to source interaction databases on problematic references that are malformed, deprecated, ambiguous or unfound. Keys for protein participants allow for retrieval of interaction information independent of the protein references used in the original records.

**Conclusion:**

We have applied our method to protein interaction records from BIND, BioGrid, DIP, HPRD, IntAct, MINT, MPact, MPPI and OPHID. The resulting interaction reference index is provided in PSI-MITAB 2.5 format at . This index may form the basis of alternative redundant groupings based on gene identifiers or near sequence identity groupings.

## Background

Protein interaction data are an increasingly important bioinformatics dataset used in biomedical research. These data are generated by a multitude of methods including both high-throughput and more traditional low-throughput proteomics studies [1] as well as *in silico *predictions based on known interactions [2].

The past several years have seen a proliferation of interaction databases as the field focuses on ways to collect these data into machine readable formats where they may be more easily computed on and reliably exchanged between users. The International Molecular Exchange (IMEx) [3] represents one effort to consolidate efforts of interaction databases by facilitating exchange of information between primary databases according to an agreed standard exchange language called the Human Proteome Organization's Proteomics Standards Initiative Molecular Interaction format (HUPO PSI-MI) [4]. Archival members of IMEx agree to share and provide a full dataset of globally available IMEx molecular interaction records (since March 31^st^, 2006) in a manner similar to the International Nucleotide Sequence Database Collaboration (INSDC) [5]. IMEx also serves to coordinate curation tasks between the partner databases to avoid redundant efforts. IMEx is open to new members and is comprised of five active partners including DIP [6], IntAct [7], MINT [8], MPact [9] and BioGRID [10].

The potential for new IMEx members is enormous: one recent compilation of protein-protein interaction resources listed over 90 databases [11]. This large list represents a lively interest in interaction data; it also represents a problem for the user searching for information since there is no unifying index.

We set out to create such an index with two goals in mind; first the index should be capable of grouping together equivalent protein interactions into a single group. The measure of equivalence should be based on exact sequence matches of the protein participants according to the source record without further interpretation based on, for example, encoding genes or near sequence identity. Second, operations performed to map protein and interaction records to these redundant groups should be preserved; this would allow for the mapping to be recreated for data integrity checking and would help identify and classify potential problem records.

Presently, IMEx archival databases provide a complete set of records generated by its members; however, these records are meant to be archival and do not resolve redundancies between records. This becomes problematic when trying to compile a non-redundant list of interactors for a given protein especially when records may use different identifiers to describe the same protein. A number of recent studies have addressed this issue and reported on databases and/or software that aim to generate consolidated interaction data sets from primary interaction databases (MiMI [12], PIANA [13] and cPath [14]). MiMI groups together redundant protein interactors and redundant interactions using "keyless identity functions" and "Deep Merging"; however, these methods are never explicitly defined [12]. PIANA software also allows for integration of protein interaction data from multiple sources and apparently resolves redundancies between different protein identifier types; again, the methods used to do this are never explicitly defined [13]. cPATH software also allows users to integrate protein interaction data from multiple sources. Redundant proteins are identified using lookup tables that may be defined by the user [14]; however, identical interactions and complexes are not grouped together. The solution presented in this study is unique in that it uses a well-defined, reproducible method to assign distinct identifiers to each distinct protein and to each distinct interaction and/or complex in which the protein participates. Our method is also unique in that it was designed to trace this process and provide feedback to source databases on problematic assignments.

**Figure 1:**
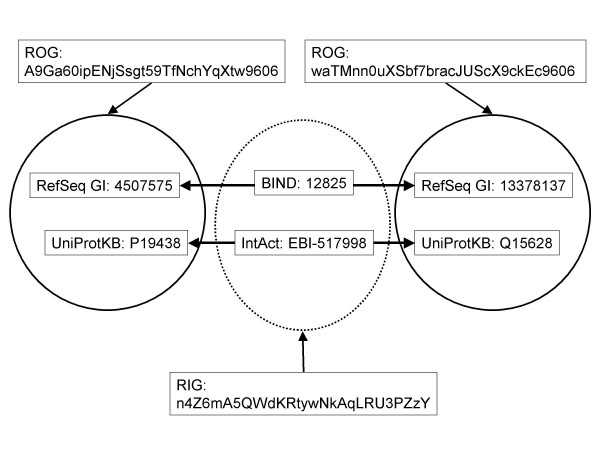
**ROG's and RIG's**. The two black circles represent redundant object groups (ROG's). Each ROG contains a set of protein sequence accessions that point to records describing the exact same sequence from the same organism. The dotted circle represents a redundant interaction group (RIG). This RIG contains a set of protein interaction accessions that point to records describing interactions between the same two proteins (ROG's). Unique identifiers for ROG's and RIG's can be calculated independently by anyone using the primary sequences of the proteins, their taxonomy identifiers and the SHA-1 algorithm.

Mapping protein identifiers to redundant groups is at the heart of this task and a number of solutions exist (see [15-19] and Figure 1). One such solution, the SEGUID database [20,21], creates and records unique identifiers (SEquence Global Unique IDentifiers) for over 6 million distinct proteins based on their primary amino acid sequence using the Secure Hash Algorithm digest (SHA-1). Two protein sequence records that describe proteins with the same primary sequence will have the same unique SHA-1 identifier and will therefore be assigned to the same redundant group. We chose to employ this existing method and resource as the basis of mapping protein identifiers to redundant groups for a number of reasons. First, the definition of redundancy is based solely on sequence. This forms a well-defined, baseline solution that is not subject to interpretation. Second, anyone can generate these keys using only sequence data and an algorithm (hence the term *global *in SEquence Global Unique IDentifiers). This facilitates accessibility to the solution. Third, the SHA-1 algorithm provides for 1.5E48 possible unique keys. This number is significantly higher than other digest algorithms (such as CRC64 and MD5; 1.8E19 and 3.4E38 keys respectively) and therefore provides further protection against collisions (i.e., two *different *protein sequences with the same key).

Our construction of a protein interaction index involved parsing PSI-MI files provided by nine interaction databases including BIND [22,23], BioGRID [24], DIP [25], HPRD [26,27], IntAct [28,29], MINT [30], MPact [31], MPPI [32] and OPHID [33]. Mapping proteins (and interactions) to redundant groups employed SEGUID-based keys and required addressing a number of issues including malformed and deprecated identifiers, incorrectly assigned taxonomy identifiers and resolution of ambiguous mappings. Methods used to make these assignments were recorded allowing for a detailed examination of potential problems with source records and our own system.

This paper describes the software system and the consolidated interaction dataset that it constructs. We demonstrate the utility of the data set and argue that the method is suited well to integrating high-throughput proteomics studies with existing interaction knowledge. We also suggest that the resulting index could provide useful feedback and search capabilities to source databases. Interaction data that may be publicly redistributed under the license agreement of the source database is indexed by the iRefIndex (interaction reference index) and made freely available in taxon-specific divisions via anonymous FTP in the PSI-MITAB 2.5 tab-delimited, text format.

## Results

The non-redundant index of interactions was constructed in four steps summarized here and described further in the following sections. First, SHA-1 digest sequence identifiers (SEGUID's) for proteins were compiled from several sources and were cross-referenced with the source database and most recent accession and taxonomy identifiers for the protein sequence record. Second, interaction data were compiled from several sources and compiled in a single relational database. Third, for each protein interactor in each interaction record, a redundant object group (ROG) was assigned using the SEGUID and taxonomy identifier for the protein. Fourth, each protein-protein binary interaction or complex was assigned to a redundant interaction group (RIG) on the basis of the ROG assignments made above. The number of distinct interaction records was examined for each source database and for each of several taxons.

### 1. SEGUID's

Sequence Globally Unique Identifiers (SEGUID's) [20] were employed to provide a unique key for each protein in the interaction dataset that was independent of the source database and accession used to describe the protein. This key may be derived by external groups using only the primary amino acid sequence and the algorithm described below. This key was used to map protein accessions used in interaction records to redundant groups and, in turn, group together redundant interaction records.

The algorithm for the creation of a SEGUID has been described previously [20]. Briefly, an amino acid sequence in single-letter code is converted to upper case after all non-letter characters and trailing or leading spaces are removed. The Secure Hash Algorithm[34] (SHA-1) is used to construct a 160-bit message digest of this amino acid sequence; we used the java.security. Message library implementation of SHA-1. This digest was converted to the base64 representation using the Base64 Java Class (Robert Harder) [35]. All trailing "=" characters used for padding were removed to yield the final 27 character long SEGUID string. SEGUID's may also be derived from primary amino acid sequence using the web interface and services provided by the SEGUID database [21]. In addition, pre-calculated SEGUID's and their mapping to various protein database accessions, aliases and FASTA files can be directly downloaded from the SEGUID FTP site. SEGUID's may be used to refer to a group of accessions that all refer to the same primary amino acid sequence (i.e. a redundant sequence group). Since two proteins in two different organisms may share the same sequence, we also employed a ROG (redundant object group) identifier to distinguish between identical protein sequences in different organisms. A ROG identifier consists of a SEGUID string concatenated with the NCBI taxonomy identifier [36]. So, for instance, while the proteins pointed to by accessions RefSeq: NP_313053 and UniProt:Q3YUU1 belong to the same redundant sequence group (SEGUID = 2c4yjE+JqjvzYF1d0OmUh8pCpz8) these proteins belong to different ROG's (ROGID's 2c4yjE+JqjvzYF1d0OmUh8pCpz8386585 and 2c4yjE+JqjvzYF1d0OmUh8pCpz8300269 respectively).

### 2. Initial compilation of molecular interaction data

Interaction records were retrieved from nine different sources and a number of elements from each record were parsed to a relational database (see Table 1 and Methods). In total, 831,760 records were retrieved including 731,656 records where all described interacting elements ("interactors") were proteins. The elements parsed from each record included the source interaction database and accession for the interaction record (where available). Each interaction record typically contains two interactors (binary interaction); however, many interaction records describe complexes where greater than two interactors are present. In these cases, the record indicates that the interactors are all participants in some complex without specifying the individual binary interactions present between the interactors. Other interaction records contain only one interactor; these records represent multimeric complexes containing only one subunit type that is present in two or more copies. In all cases, the interactors present in an interaction record were examined and an entry was made for each in the relational database. No attempt was made at this point to consolidate redundant interactors; every interactor in every interaction record is considered a unique object in the relational database. The entry for each of these interactors included:

**Table 1:** Molecular interaction data sources incorporated by iRefIndex.

**Source**	**Format**	**Location**	**Version (date)**
BIND	Tab-delimited text file.	/(see Methods). 20050525.complex2refs.txt 20050525.ints.txt 20050525.refs.txt 20050525.complexes.txt 20050525.labels.txt 20050525.complex2subunits.txt	May 25, 2005.
BioGRID	PSI-MI 2.5	BIOGRID-ORGANISM-2.0.38.psi25.zip	Version 2.0.38
DIP	PSI-MI 2.5	dip20080114.mif25.gz	Jan. 14, 2008
HPRD	PSI-MI 2.5	HPRD_SINGLE_PSIMI_090107.xml.tar.gz	Release 7. Sept. 1, 2007
IntAct	PSI-MI 2.5	pmidMIF25.zip	Mar. 15, 2008
MINT	PSI-MI 2.5	full.psi25.zip	Dec 21, 2007
MPact	PSI-MI 2.5	mpact-complete.psi25.xml	April 19, 2007
MPPI	PSI-MI 1.0		Not available
OPHID	PSI-MI 1.0		July 18, 2006

1) a single primary reference describing the interactor in an external sequence database,

(PSI-MI 2.5 Path: entrySet/entry/interactorList/interactor/xref/primaryRef)

2) a list of secondary references that may also point to the interactor in an external sequence database, (PSI-MI 2.5 Path: entrySet/entry/interactorList/interactor/xref/secondaryRef)

3) an NCBI taxonomy identifier describing the source organism of the interactor,

(PSI-MI 2.5 Path: entrySet/entry/interactorList/interactor/organism) and

4) the primary amino acid sequence of the protein interactor,

(PSI-MI 2.5 Path: entrySet/entry/interactorList/interactor/sequence).

Only the first element is mandatory under the PSI-MI schema. The other three elements were retrieved and recorded whenever present. All four elements were used during the next stage of processing in an attempt to map the interactor to a redundant object group (ROG).

### 3. Mapping protein interactors to redundant object groups (ROG's)

**Figure 2:**
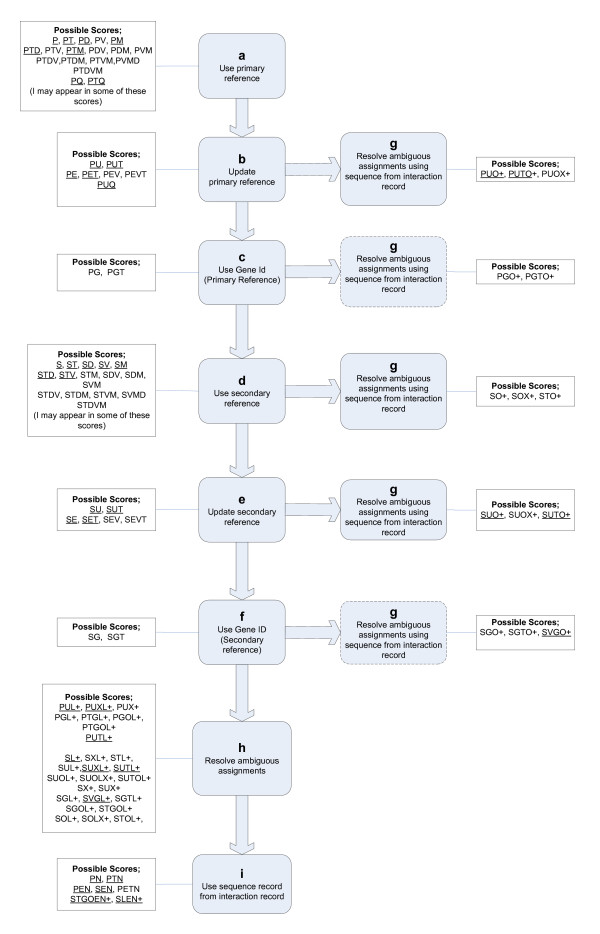
**Overview of the ROG assignment process**. Logical blocks (a-i) are described in the text. Only the assignment scores shown in this diagram are reachable by the workflow. Underlined scores were obtained for the present build of the database. A key explaining assignment score features is given in Table 2.

The data retrieved for each interactor from each interaction record were used in the process outlined in Figure 2 to assign a ROG identifier to each protein interactor. This process follows an order of preference for the data used to make the ROG assignment. An attempt at assignment is first made by using the primary reference provided in the interaction record. If this fails, the secondary references provided in the interaction record are used. If this fails, the protein's primary amino acid sequence is used (if provided in the interaction record). The process ends as soon as a non-ambiguous assignment is made.

In the course of this assignment, a ROG "assignment score" is generated; this is a nominal value that provides information on how the assignment was made. The assignment score is composed of a set of features that may each have a value of 1 or 0. A human readable version of this score is generated by representing each feature with a value of 1 with a single character unique to that feature. These characters and their mappings to assignment score features are listed in Table 2. As an example, the score represented by "PUO+" indicates that the assignment was made using the primary accession (P) from the interaction record, that the accession required updating (U), and that there were more than one (+) possible updated accessions but only one of these accessions pointed to a sequence that was identical to the original (O) sequence listed in the interaction record. In effect, assignment scores are associated with a path through our workflow. A number of scores are possible when using our workflow. These possible scores are listed in Figure 2 and scores that were actually observed are underlined.

**Table 2:** Features of the ROG assignment score and their corresponding character representations.

**Character**	**Description of feature (when the value is 1).**	**Frequency**^1^
P	The *interaction record's *primary (P) reference for the protein was used to make the assignment.	79.196

D	The source database (D) listed in the interaction record is different than what is expected for the given accession for the protein. In specific cases, this difference is tolerated and the assignment is made.	3.167

T	The taxonomy (T) identifier for the protein (as supplied by the interaction record) differed from what was found in the protein sequence record. This discrepancy was tolerated and the assignment was made.	16.134

M	The protein reference listed by the interaction record was a typographical modification (M) of a known accession. In specific cases, this variation is tolerated and the assignment is made.	0.289

V	The protein reference listed by the interaction record contained version (V) information that was ignored. For example, RefSeq accession.version NP_012420.1 was listed but treated as RefSeq accession NP_012420.	0.591

Q	The protein reference used to make the assignment was of the type "see-also". See PSI-MI Path: entrySet/entry/interactorList/interactor/xref/primaryRef/refType = "see-also".	0.003

U	The protein reference listed in the interaction record and used to make the assignment was a secondary UniProt accession and was updated (U) to a primary UniProt accession in order to make the assignment.	2.817

E	The protein reference was a retired NCBI Identifier. NCBI's eUtils (E) were used to retrieve the current accession and/or sequence.	1.537

I	The protein reference used was an NCBI GenInfo Identifier (I).	1.741

G	The interaction record's reference for the protein was an EntrezGene (G) identifier. The corresponding products of the gene were used to make the assignment.	0.581

S	One of the *interaction record's *secondary (S) references for the protein was used to make the assignment.	20.804

+	More than one possible assignment is possible (+). This case may arise in one of three ways. 1) The reference supplied by the interaction record requires updating but more than one possibility exists. For example, Q7XJL8 was found to be a secondary accession in three separate UniProt records (Q3EBZ2, Q6DR20, and Q8GWA9). 2) The secondary references supplied by the interaction record point to more than one unique protein sequence. 3) An EntrezGene identifier is provided in the interaction record as a protein reference. This identifier points to more than one protein product. An attempt is made to resolve this ambiguity as indicated by ROG score features O, X or L (see below).	1.296

O	More than one possible assignment is possible (see + above). The assignment chosen has a SEGUID that is identical to the SEGUID of the original (O) sequence provided in the interaction record.	0.469

X	More than one possible assignment is possible (see + above). The assignment chosen has the same taxonomy (X) identifier as listed in the interaction record.	0.002

L	More than one possible assignment is possible (see + above). The assignment with the largest (L) SEGUID is arbitrarily chosen (see Methods).	1.259

N	The protein reference, taxonomy identifier and sequence for the protein as provided in the interaction record are used to make a new entry in the SEGUID table. The protein interactor is assigned the newly (N) generated ROG identifier.	2.024

^1^Total number of assignment scores where this occurs expressed as a percentage of all assignments (774,086).

#### 3.1 Detailed description of the assignment process

Each logical block in the assignment process is represented in Figure 2(a–i) and is further described below.

#### a: using the primary reference

The assignment process begins with a consideration of the primary protein reference given by the interaction record. The reference consists of an external database identifier (e.g. UniProt) and an accession pointing to a record in that database (e.g. P31946). The taxonomy identifier for the protein is also considered. If the accession is found in the UniProt partition of the SEGUID table with the expected taxonomy identifier, then the protein interactor is assigned the corresponding SEGUID and ROG identifiers with an assignment score of "P". Seventy nine percent of all assignments were made using the primary reference (see Table 2). The external database or taxon provided by the interaction record may be ambiguous (e.g. "protein accession" is listed as the database or "mammalia" is listed as the taxon). In these cases, database (D) and/or taxon (T) criteria may be relaxed in searching the SEGUID table; the assignment is made with the corresponding score (PD, PT or PTD). Unexpected database names are only tolerated when the provided protein accession is alphanumeric. Unexpected taxon identifiers are always tolerated. In all cases, these discrepancies are recorded along with the expected database name and/or taxon. In some cases, the accession found in the interaction record may require modification in order to find a matching entry in the SEGUID table (see Methods). For example, minor typographical changes are allowed (NP 012420 is allowed to match NP_012420) or the version number of an accession is ignored (NP_777219.1 is allowed as a match to NP_777219). These cases are indicated by score characters M and V respectively. Lastly, protein references are associated with a "type" that may be either "identity" or "see also". The later indicates that the reference does not point to a record about the interactor's sequence but to a record where additional information can be retrieved about the protein. In a few rare cases, the primary reference was of the type "see also". Assignments were made with these references and marked by the score character Q (see Table 2).

#### b: updating accessions

Protein sequence records from UniProt may be altered over time. In some cases, the new record will receive a new primary accession and the old accession will be included in the new record as a secondary accession. It is also possible that a sequence record may be used to make more than one new sequence record. In these cases, the old accession will appear as a secondary accession in each of the new records [37]. Our work-flow checks for these cases. In the event that a UniProt accession for a protein (as provided in the interaction record) is not found in the SEGUID table, an attempt is made to update the accession. This is accomplished by searching for UniProt records that list the interaction-record-provided accession as a secondary accession. If only a single record is found, the UniProt primary accession in this record is taken as the updated accession. The corresponding assignment score will contain a U (see Table 2). On the other hand, it is possible that more than one record is found and that these records describe different protein sequences (ROG's). In this case, the mapping from the interaction-record-provided accession to an updated accession is said to be ambiguous. This ambiguity may be resolved in block g using the protein's sequence when provided by the interaction record (see below).

NCBI *accessions *do not require an analogous logical block. An NCBI accession follows a sequence record for its lifetime. Changes in the sequence are indicated by changes to its version number and its primary GenInfo Identifier (GI) [38,39]. The assignment process considers NCBI accessions and will ignore version information where provided (the assignment score contains a V). In the case that GI's or Protein Databank identifiers are provided, Entrez Programming Utilities (eUtils) [40] are used to retrieve the GenBank or RefSeq *accession*. Corresponding assignment scores will contain the letter I to indicate a GI was used and the letter E to indicate that eUtils were used. In summary, an attempt is always made by the assignment process to update the protein's reference to its most recent version.

#### c: using gene identifiers

In the event that the primary accession is still not found in the SEGUID table and the accession provided is an Entrez GeneID [41], the protein accessions corresponding to the gene are retrieved. In the best case, only one protein product is present and its taxonomy identifier matches the one given in the interaction record; the assignment score will contain a G. This block also allows for a relaxed taxon match during search of the SEGUID table (score contains a T). In the event that more than one protein is encoded by the gene, the assignment may be made using the protein's sequence when provided by the interaction record (similar to g-block code).

#### d, e, f: using secondary references

In those cases where an assignment cannot be made using the primary reference provided by the interaction record, the secondary references are consulted. Corresponding assignment scores will contain the letter S. These three blocks of code essentially follow the same logic as their analogous blocks for the primary reference (a, b, c). Only those secondary identifiers with the type "identity" are considered in these steps. These blocks also account for the possibility that the set of secondary references may point to more than one protein (ROG). This ambiguity may be resolved in block g using the protein's sequence.

#### g: resolving ambiguities using interaction record provided sequence

This logical block attempts to resolve ambiguous assignments that may have arisen in the above blocks by using the protein sequence (as provided by the interaction record). This case is marked in the assignment score by the character "+" and may be resolved if the SEGUID for the sequence (provided by the interaction record) matches one (and only one) of the possible assignments (see O in Table 2).

#### h: resolving ambiguities using arbitrary methods

This logical block makes an arbitrary assignment where more than one assignment is possible and the ambiguity could not be resolved by using the protein sequence (block g). This case is marked in the score by the character "+" and may be arbitrarily resolved by choosing the assignment that has the expected taxonomy identifier (X) or by simply choosing the assignment with the largest SEGUID (L). The largest SEGUID is determined as the last in a list of SEGUID strings that have been sorted in ascending lexicographical order (see Methods). Arbitrary assignment is a stop gap measure and assignments with scores containing L or X (without an O) should be treated with caution (see X and L in Table 2).

#### i: using interaction record sequences and archival sequences

In the event that no assignment is possible (i.e., no matching entry is found in the SEGUID table), but the interaction record lists a sequence, the SEGUID is calculated for the sequence and a new entry is made in the SEGUID table (see N in Table 2). The interactor is assigned to the corresponding ROG. Likewise, if a protein reference can be used to retrieve an archived (obsolete) sequence from eUtils, the SEGUID is calculated for the sequence and a new (N) entry is made in the SEGUID table. This mechanism serves as a stop-gap measure (see section 3.3).

#### 3.2 Review of assignments made by database

Table 3 details the number of protein interactors that could be assigned to redundant object groups (ROG's) broken down by data source. Approximately, 96% of all protein interactors were unambiguously assigned to a ROG with some score (column 4). Scores associated with some ambiguity accounted for only 1.2% of the total number of interactors (column 5). In total, 21451 protein interactors (2.8%) could not be assigned to a ROG present in our starting SEGUID table (columns 6 and 7). Reasons for this failure varied. In some cases, interaction records referenced proteins using non-protein identifiers such as PubMed, DNA and mRNA identifiers. In other cases, protein references included accessions for protein data repositories not in our starting SEGUID table (for example, MIPS, EBI, UniParc and MINT). Retired accessions that could not be mapped to a new accession also fell into this class (e.g. RefSeq: XP_234709). In those cases, where a sequence was provided with the interaction record or an archived (obsolete) sequence could be retrieved, a new (N) entry was made in the SEGUID table (column 6).

**Table 3:** Assignment of protein interactors to ROG's.

**Source**^1^	**Intrctrs**^2^	**Assigned**^3^	**%**^4^	**Arb.**^5^	**New**^6^	**Unassigned**^7^	**Unique proteins**^8^
BIND	285482	273199	95.7	0	8389	3894	40744
BioGrid	25845	17047	66.0	8742	3	53	25205
DIP	19585	18257	93.2	400	380	548	18934
HPRD	90939	90516	99.5	307	108	8	9540
IntAct	86094	82530	95.9	15	3364	185	38407
MINT	80543	77179	95.8	1	2881	482	28084
MPACT	40349	40112	99.4	0	0	237	4972
MPPI	3628	3281	90.4	178	33	136	858
OPHID	146423	145587	99.4	86	491	259	9710
ALL	778888	747708	96.0	9729	15649	5802	77827

^1^Interaction data source (see methods).

^2^Total number of interactors found in all interaction records regardless of type (includes redundant entries).

^3^Number of proteins assigned unambiguously to a ROG. Assignments listed in columns 5 and 6 are not included here.

^4^Column 3 expressed as a percentage of column 2.

^5^Total number of ROG assignments that were ambiguous and resolved with an arbitrary method (see ROG scores with 'L' in Figure 2).

^6^Total number of assignments made where a new sequence was added to the SEGUID table (see ROG scores with 'N' in Figure 2).

^7^Total number of protein interactors that could not be assigned to a ROG.

^8^Total number of unique proteins (ROG's).

#### 3.3 Review of assignment scores

Table 4 details the number of protein references that could be assigned to redundant object groups (ROG's) broken down by assignment score. The assignment process resulted in over 50 different scores describing how each assignment was made. A subset is shown with examples. To facilitate analysis, we have grouped these assignment scores into six types (column 1).

**Table 4:** Number of protein references successfully assigned to ROG's and broken down by assignment score.

		Examples		
		
Score type	Total number with this score type (%)	ROG Assignment Score	Number of cases	Details for one example
1	598590 (77.43)	P	512650	UniProt:Q15118 is cited in the interaction record as the primary reference (P).
		S	52738	UniProt:P94102 is cited in the interaction record as the secondary reference (S).
		PD	14166	"protein accession" is cited as the source database for accession Q9Z2F5 (D).
		SM	2154	Accession NP 191913 is cited in a modified form (M) without the underscore.
		SVGO+	262	EntrezGeneId:26207 (G) encodes multiple proteins (+) but only one matches the original (O) sequence given in the interaction record (RefSeq:NP_858057.1).

2	24664 (3.19)	PU	18542	UniProt:O95686 is cited and updated (U) to UniProt/KB:Q9UQK1.
		PE	264	GenBank GI:12962935 is cited and updated to RefSeq:NP_002458.2 using eUtils (E).
		PUO+	6	UniProt:P38706 is cited. Two possible updates are possible (+) but only one matches the original (O) sequence in the interaction record (P0C2H6).

3	121540 (15.72)	PT	52074	Protein reference cites taxon id as 9534 (African green monkey) but the sequence record cites taxon 9606.
		ST	60205	Protein reference cites taxon id as 40674 (mammalia) but the sequence record cites (9606) human.

4	2803 (0.36)	PTUO+	15	UniProt:O04063 is cited with taxon identifier 4530 (rice). More than one updated accession exists (+U). Only one possibility has the same sequence as cited in the interaction record (P0C5B0) with taxon identifier 39947 (a specific strain of rice).

5	9840 (1.27)	SL+	9090	The primary reference cited is not found. 49 secondary references are cited (S). 15 of these were found to map to 8 distinct proteins (+). The protein with the largest (L) SEGUID is arbitrarily chosen.
		PUTL+	187	UniProt:Q9MAY7 is cited with a taxon id of 4530 (rice). Two updated accessions are available (+U). Neither one has the expected sequence or taxon id (T) given in the interaction record. The accession with the largest (L) SEGUID is arbitrarily chosen.
		SVGL+	303	EntrezGene:9912 is cited (G). This gene encodes two proteins (+). Neither has the sequence expected from the interaction record. The one with the largest (L) SEGUID is selected.
		PTQ	21	Primary accession P84244 cited as a "see also" (Q) reference with taxon id 9606. The sequence record cites taxon id 10090 (T).

6	15649 (2.02)	PN	8909	Q95Q01 is an obsolete accession. The sequence is retrieved from the interaction record. The SEGUID and ROGID are calculated and stored locally as a new entry (N).
		SEN	5561	RefSeq:NP_010441 is an obsolete accession. The sequence is retrieved using eUtils (E). The SEGUID and ROGID are calculated and stored locally as a new entry (N).
		STGOEN+	2	EntrezGene 196549 (G) is cited and encodes two proteins (+). The protein accessions cited by EntrezGene are retired. Sequences are retrieved using eUtils (E). One matches the sequence cited in the interaction record (O). The SEGUID and ROGID are calculated and stored locally as a new entry (N).

Type 1 assignments were least problematic. In all cases, an unambiguous assignment to a ROG was possible using either a primary or secondary reference (P, S). In a few cases, version information was ignored (V), the source database (D) was relaxed or minor modifications (M) to the accession were allowed in order to find the corresponding entry in our SEGUID table. In total, type 1 assignments accounted for 77% of the assignments made.

Type 2 assignments required that the accession provided by the source database be updated using either UniProt secondary accessions (U) or NCBI eUtils (E). In all cases, an unambiguous assignment was made. In a few cases, the sequence provided by the interaction database was required to accomplish this (score PUO+). In total, type 2 assignments accounted for 3% of the assignments made. There is likely always to be some asynchrony between interaction database releases and the major sequence databases; the ability to map accessions to their most recent versions is therefore an essential component of any integrative effort.

Type 3 assignments involved references where the taxonomy identifier provided by the interaction database was different than the 'true' taxon provided by the source sequence record. Type 4 assignments represent those rare cases where both an update to an accession was required and the true taxonomy identifier was different than expected. Type 3 and 4 cases accounted for 16% of all assignments and were typically the result of the interaction record listing a taxon that is parental to the true taxon (e.g. mammalia is listed in place of human or a species taxon is used in place of the 'true' sub-strain identifier). For the most part, this practice was not a problem for our purposes because the protein reference pointed to a single sequence record where the 'true' taxonomy identifier was listed. However, these differences must be carefully considered when analyzing data or when designing search strategies based on taxonomy identifiers.

Type 5 assignments involved references that could be mapped to a number of different proteins (see + in assignment score) and that could not be resolved using sequence data provided in the record. In some cases, this was resolved by choosing the ROG that had the expected taxonomy identifier (X) or by the arbitrary method of choosing the assignment with the largest SEGUID (L) according to its ASCII value. The majority of cases arose from use of internal identifiers for the protein's primary reference and where the list of alternative secondary accessions pointed to multiple proteins. PSI-MI guidelines suggest that proteins be represented with stable identifiers such as UniProtKB or RefSeq accessions [4] and our results would recommend that distribution of internal identifiers in PSI-MI files be avoided. This would have been possible in the majority of cases. In a few cases, retired UniProt accessions were found that had been split into several new records. The correct mapping was not discernable even when taking into account taxon and sequence information present in the interaction record (see scores with characters U, L and +). Ambiguity also arose from the use of EntrezGene identifiers that point to multiple protein products.

Finally, type 6 assignments involved interactors for which no matching reference or sequence existed in our SEGUID table. The protein sequence provided by the interaction record (or retrieved from archival sources) was used to construct a new (N) SEGUID entry. This served to group together any other interactors that might have the same sequence in the current build of the index. This is a stop-gap measure and new SEGUID entries are discarded from one build of the database to another. The majority of these cases are due to NCBI *accessions *that have been retired and for which mappings to new accessions are non-existent and not easily automated (e.g. RefSeq: NP_116649). This discontinuity between related sequences is a limitation of NCBI accession use. In some cases, UniProt accessions circumvent this problem by providing secondary (retired) accessions in active records as a built-in history of the sequence; however, in cases where sequence records are split, this may lead to multiple mappings that are not easily disambiguated (see above). On the other hand, the NCBI accession.version and GenInfo system allows for simple and unambiguous updating of a sequence while the accession is active (BIND uses this system and had no ambiguous (type 5) assignments). In the end, there is no perfect system and integrating data will be dependent on updates and clarifications from source databases.

In summary, our method has allowed us to unambiguously map 96% of all protein interactors to redundant object groups (Table 4, score types 1–4). The remaining 4% of proteins are problematic either because our mappings are ambiguous (score type 5) or because we are unable to make any mapping at all to a current sequences (score type 6 and unassigned). These identifiers will be the subject of further investigation with the source databases. The Protein Identifier Cross-Reference Service [16] was released while this project was under development; implementation of this resource provides access to UniParc sequences and may resolve some of these unassigned identifiers. Table 4 results have been broken down by database and will be made available to interested source databases.

#### 4. Mapping protein-protein binary interactions and complexes to redundant interaction groups (RIG's)

Each interaction record involving only protein interactors was assigned to a redundant interaction group (RIG) on the basis of the ROG assignments made above. A RIG identifier (RIGID) is constructed by concatenating ROG identifiers (after sorting them in ascending lexicographical order), applying the SHA-1 algorithm to the resulting string, converting the digest to its base64 representation and removing all trailing "=" characters used for padding (see Methods). The RIGID constitutes a unique and universal pointer to a set of interaction or complex records that all involve the same proteins from the same organism. Table 5 summarizes our efforts to map source interaction data to redundant interaction groups. In total, we collected 731,656 interaction records that involved only proteins. 99% of these were assigned ROG's for each interactor and the corresponding RIG was calculated. In total, we found 318,349 RIGID's corresponding to distinct interactions. 293,216 (92.1%) of these corresponded to binary protein interactions, 11,128 (3.5%) to complexes involving 3 or more proteins and 14,005 (4.4%) to interactions involving only one interactor type (multimers). Only 44% of the RIGID assignments represented distinct interactions or complexes. This redundancy in the consolidated dataset was higher than the redundancy within any given source (column 5) suggesting that there was a fair amount of redundancy between datasets. We examined pairs of source datasets (Table 6) and showed that this was the case. There were no examples of one data source completely subsuming another. In fact, each source possesses a large number of distinct interactions. This further illustrates the need for a consolidated dataset. At the same time, this demonstrates that a user searching for interaction information using a number of web-site interfaces would be overcome with redundant interactions; many of these redundancies are not apparent by a visual inspection of the records especially when different protein identifier schemes are used.

**Table 5:** Summary of mapping interaction records to RIG's.

**Source**^1^	**Total records**^2^	**Protein-only interactors**^3^	**PPI Assigned to RIGID**^4^	**Unique interactions**^5^
BIND	193648	93957	91275 (97.15)	62893 (68.9)
BioGRID	204440	204440	204247 (99.91)	132076 (64.7)
DIP	56314	56314	55105 (97.85)	54951 (99.7)
HPRD	76145	76145	76133 (99.98)	37885 (49.8)
IntAct	104791	104378	104052 (99.69)	91669 (88.1)
MINT	104847	104847	103474 (98.69)	73693 (71.2)
MPACT	16504	16504	16286 (98.68)	13321 (81.8)
MPPI	1814	1814	1695 (93.44)	827 (48.8)
OPHID	73257	73257	72907 (99.52)	47338 (64.9)
**ALL**	831760	731656	725174 (99.11)	318349 (43.9)

^1^Interaction data source (see methods).

^2^Total number of interaction records found in source.

^3^Total number of interactions involving only protein interactors.

^4^Number of interactions where all protein interactors were assigned to a ROG (score types 1 to 6). Percentage of column 3 is shown in brackets.

^5^Number of unique protein interactions and complexes (RIGID's) found in the data source (expressed as a percentage of column 4 in brackets).

**Table 6:** Redundancy between pairs of interaction datasets processed in this study.

BIND	**62893**								
BioGRID	20929	**132076**							
DIP	25969	28314	**54951**						
HPRD	2766	2397	725	**37885**					
IntAct	25172	21658	25728	7785	**91669**				
MINT	23303	32018	31484	6774	41580	**73696**			
MPACT	6837	8416	6744	0	5983	6458	**13321**		
MPPI	362	15	26	278	71	101	0	**827**	
OPHID	2124	1673	789	17611	6902	7013	0	168	**47338**
	BIND (25481)	BioGRID (83495)	DIP (12131)	HPRD (15638)	IntAct (41324)	MINT (15409)	MPACT (1139)	MPPI (277)	OPHID (26854)

Each cell represents the number of unique interactions shared between two data sources. Numbers in brackets represent the number of interactions only found in one database. Bold numbers represent the number of distinct interactions and complexes (RIGID's) in a given dataset. For example, 62893 distinct interactions and complexes were found in BIND and 25481 of these could only be found in BIND. BIND and DIP shared 25969 distinct interactions and complexes.

Table 7 breaks the number of unique RIGID's down by organism. Yeast appears to be the most intensively documented species with a RIG to ROG ratio of 14.2 followed by humans which hold the highest number of interactions in this data set.

**Table 7:** Number of unique RIG's and ROG's by source organism.

**Organism**	**Taxon**^1^	**Unique RIG's**	**Unique ROG's**	**RIG/ROG ratio**
*Homo sapiens*	9606	112466	21615	5.2
*Mus musculus*	10090	11173	6822	1.6
*Rattus norvegicus*	10116	3656	2635	1.4
*Caenorhabditis elegans*	6239	11090	4757	2.3
*Drosophila melanogaster*	7227	44707	10574	4.2
*Saccharomyces cerevisiae*	4932	89498	6293	14.2
Other	All others	56486	26896	2.1

^1^At least one interactor in the counted RIG's has this taxonomy identifier according to either the source sequence record or source interaction record.

### Utility of consolidated data

**Figure 3:**
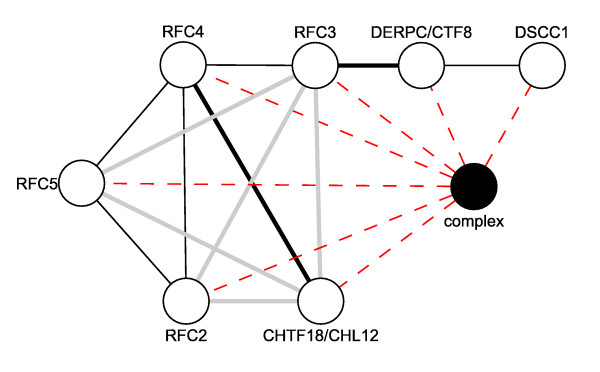
**Utility of consolidated interaction data**. The black node represents a complex record describing the alternative Ctf18-Dcc1-Ctf8-replication factor C complex (see HPRD complexes for EntrezGene 63922 and [42]). Open circles represent proteins. Dashed red lines represent membership of a protein in the complex. Solid lines represent binary protein-protein interactions that are found only in HPRD (thick black lines), only in OPHID (thick grey lines), or in more than two databases (thin black lines). The clustering coefficient of the complex is 0.57.

A great deal of interaction data is derived from high-throughput methods based on co-immunoprecipitation with a tagged bait protein. These data result in "complex" interaction records with many interactors where the interactions between specific protein subunits remain unknown. Consolidation of these complex data with pair wise interaction data allows for the development of a more complete picture of the potential biological complexes. Figure 3 shows a bi-partite graph where a complex record is represented with a closed circle and protein members of this complex are indicated by open circles and adjoining edges to the complex node. The clustering coefficient of this complex will approach 1 as all potential interactions between members are met by pair wise interactions from the consolidated database. The complex represented is the alternative Ctf18-Dcc1-Ctf8-replication factor C complex and was observed using affinity purification methods [42]. Evidence for 12 of the 21 pair wise interactions between its protein members is provided by 4 interactions databases including 5 interactions found only in OPHID and 2 interactions that are only found in HPRD. This example illustrates the utility of combining data from multiple sources while treating pair wise and complex interaction data as separate but complementary data types. This subject has been explored in detail with methods proposed by Scholtens and Gentleman demonstrating how data from affinity purification and yeast two-hybrid experiments can be combined to form a more complete picture of potential complexes [43,44]. We suggest that data from the iRefIndex would be amenable to this same type of analysis since it includes both complex and binary interaction data in a single non-redundant index.

**Figure 4:**
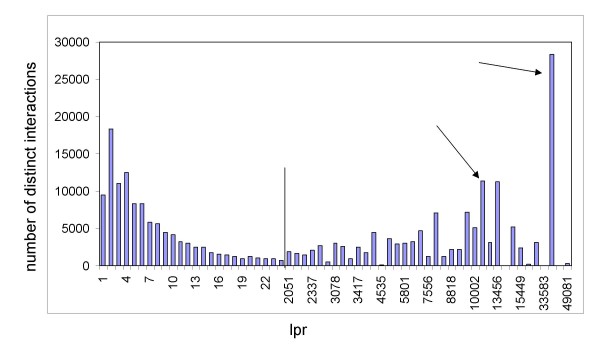
**Distribution of PMID re-use amongst interaction records**. Distinct interactions are binned according to "lowest pmid re-use" (lpr). Bars indicate the number of distinct interactions (RIG's) with a given lpr. The body contains a long distribution of interactions with lpr values between 25 and 2050 (omitted and indicated by the vertical line). Not all lpr bins are represented after lpr of 25. The bins of interactions at lpr 11781 and 36744 (arrows) correspond to yeast two-hybrid studies of the *Campylobacter jejuni *[71] and *Drosophila melanogaster *[72] interactomes respectively.

The non-redundant nature of our data set has also allowed us to classify interactions based on supporting literature. PubMed Identifiers (PMID's) listed in interaction records point to literature references that support an interaction. A PMID may be used to support more than one interaction. The lpr score (lowest pmid re-use) is the lowest number of unique interactions that are supported by one of the interaction's PMID's. An lpr value of one would indicate that at least one of the PMID's supporting the interaction has never been used to support any other interaction and that the interaction is not likely to rely solely on high-throughput data. Figure 4 shows a distribution of interactions according to their lpr. These data show a bi-modal distribution where 38% of all distinct interactions are supported by low-throughput papers (lpr less than 25). These interactions may be taken (in a crude sense) to be more reliable than those relying solely on high-throughput data. In contrast, 46% of interactions have an lpr greater than 2050 and can be considered high throughput. The long body of the distribution contains "middle throughput" interactions (not shown in Figure 4) composing 16% of all distinct interactions.

The publicly distributable subset of the iRefIndex is available in a PSI-MITAB 2.5 tab-delimited text file under a Creative Commons license (see Methods). This format allows for the types of analyses described above.

## Discussion

This paper presents a detailed description of methods used to consolidate protein interaction data from a variety of sources. We have paid special attention to describing exactly how assignments to redundant groups have been made. Our goals have been three-fold. First, we hope that this resource will be used by source interaction databases to identify problem records and improve data accessibility. Second, we believe that a non-redundant dataset is essential to future work (especially where it involves complex data). Third, we have presented methods to generate global keys for protein interactions and complexes.

Redundancy can be defined in a number of ways. We have chosen to define ROG's based on exact sequence and taxonomy identifier match. ROG's may be further grouped into larger redundant groups that include all products derived from a single gene or proteins that are very similar in sequence. We could have based ROG's on gene identifiers to begin with; but, we do not believe that this is the best possible course for three reasons. First, not all proteins are easily mapped to Entrez Gene identifiers. Second, many genes encode redundant protein products. Finally, mapping an interaction to a set of gene identifiers suggests that all products of those genes are involved in the interaction. Once this generalization has been made, there is no way back to specify that some pairs of gene products do not interact. Additionally, the methods suggested here allow external groups to generate universal RIG's and ROG's for their own data sets and integrate them with this dataset. They may then choose to further redefine redundancy according to their own purposes.

As an example, modelling the structure of large macromolecular complexes such as the nuclear pore complex [45] is dependent on information from a collection of sources (including interaction data) that helps constrain possible structure solutions. Interaction data for complex structure components may be collected from a variety of databases using our index. Further these data may be supplemented with interaction data for analogous components in a range of organisms. These protein components may have low to near sequence similarity to the modelled components; their interactions could be easily overlaid with the modelled complex using a simple Perl script and file that maps analogous proteins into the same redundant object group. As further example, recent work has shown that genes associated with complex diseases tend to cluster in the human interaction network [46,47]. Again, these clusters could be supplemented with interaction information from analogous gene products in other organisms. Locating these interactions is facilitated using a list of sequence-related ROG identifiers since they are independent of the protein database references used to construct the original interaction records.

Our experience suggests that the casual user of interaction data would find it practically impossible to collect and consolidate interaction data using web-interfaces to the numerous source databases. In addition, the effort expended in setting up and optimizing this system suggests that the task is also inaccessible to most bioinformatics groups unless they are dedicated to analyzing interaction data (see Methods).

The present index provides useful access to a non-redundant view of interaction data using updated protein identifiers and corrected taxonomy identifiers. This paper represents more than a year of development time but is still a work in progress that will require further dialogue with the source databases. In the mean time, users are advised to keep in mind a number of cautionary points.

First, no assessment was made of the accuracy of source records. Source records and literature references should be consulted for further details. We have assumed that protein references listed in interaction records represent those interactors described in the primary literature. In truth, in some cases, the authors of this primary literature themselves may be uncertain of the identity of the exact gene products or protein isoforms that mediate the interaction. In other cases, authors may reference proteins using only a name with no database reference. There is no allowance (or prescribed method) within the PSI-MI specification to deal with such ambiguity and different databases deal with this problem in different ways. BioGRID, for instance, intentionally curates interactions at the gene level; all of the protein products for an interactor's gene may be listed within an interaction record. This practice led to a number of ambiguous assignments for BioGRID interactors. Members of the IMEx consortium adhere to published curation guidelines and we would suggest that other databases also publish their guidelines. Guidelines will understandably differ (even within the same database over time); making these guidelines transparent is an essential part of providing access to data.

Second, PSI-MI XML is a means of exchanging data. It does not guarantee uniform representation of meaning. For instance, some experimental methods (such as immunoprecipitations from cell extracts) will result in "complex data". IntAct PSI-MI XML records will represent these data as a list of multiple interactors in a single interaction. This "complex" grouping carries no information about the binary interactions between member proteins or about their stoichiometry. In contrast, BioGRID will represent complex data using a series of binary interactions with one "hub" interactor in common where the hub may be a tagged "bait" protein used to immunoprecipitate other proteins from an extract (the so-called "spoke" representation). The later representation implies binary interactions that may not exist and runs the risk of confounding complexes that share a common "hub". The IntAct method is more amenable to our bi-partite representation in Figure 4 where complexes are represented by a separate node type. These differences in representation may be accounted for during analysis or possibly even normalized prior to analysis. Again, publication of curation guidelines is an important first step towards this. At present, the iRefIndex PSI-MITAB file preserves complex information where it is present in the source record.

In the near future, we hope to collaborate with members of the IMEx consortium to create a reference index of all publicly available interaction data. The two BioGRID examples given above came to light after soliciting feedback from all source databases on our results; other examples are certain to follow. Eventually, the issues identified by this process can be built into checks carried out by the PSI-MI Validator [4,48] to avoid recurrence of these problems by databases and data submitters.

Providing a web interface to these data is an obvious priority. We believe this would be best accomplished by providing a programmatic web-services interface to our data. This would allow source databases and applications such as Cytoscape to provide a user interface to these data that would re-direct users to the appropriate source database(s). A Common Query Interface was proposed at the recent HUPO-PSI meeting in Toledo, Spain and development is in progress on its implementation by each of the databases [4].

Finally, our index has the ability to consolidate interaction records derived from a common publication by multiple source databases. This view would facilitate cross-checking between databases where such duplications exist. Further, this view may help in the process of normalizing the representation of interactions using common standards and controlled vocabulary. This process is expected to be most important for legacy records predating the 2006 IMEx consortium agreement.

## Conclusion

The iRefIndex dataset represents a carefully constructed non-redundant index of interaction data. This resource has numerous applications and may form the basis of further efforts to improve access to information and to normalize representation of interactions.

## Methods

### Construction of SEGUID tables

The SEGUID proteome database [21] was downloaded in tab-delimited format and loaded into a MySQL [49] database table with corresponding column names and data types. This data set lists SEGUID's for about 6 million proteins along with their corresponding protein database sources, accessions and taxonomy identifiers. This table was supplemented with our own source-database identifier (allowing us to map the provided source database names to a normalized list of databases provided by the PSI-MI database-citation controlled vocabulary [50]). In addition, we added a Redundant Object Group (ROG) identifier and unique integer equivalents for each distinct SEGUID and ROG identifier to facilitate faster record retrieval on an integer key.

We independently regenerated SEGUID entries for recent releases of UniProt (Release 13.1) [51] and RefSeq [52] (Release 28). UniProt SEGUID's were regenerated in order to resolve SEGUID differences between GenBank [53] and UniProt versions of the same accession due to asynchrony between the two databases at the time. GenBank's versions of UniProt sequences were deleted from the original SEGUID dataset to prevent this problem from recurring. Protein isoform sequences were also retrieved for UniProt sequences and their corresponding SEGUID entries were made. Finally, we independently regenerated SEGUID entries for GenBank sequence records derived from the Protein Data Bank's (PDB)[54] structural records in order to retrieve chain identifiers for protein accessions. These suggested modifications have been relayed to the SEGUID database.

Calculation of SEGUID's for approximately 3.8 million RefSeq records required 32 minutes using the hardware configuration described below. This included the time required to read sequence from a database, calculate the SEGUID and write the results to a data table.

The final SEGUID table was partitioned into five tables according to the source database in order to facilitate lookup and retrieval time. These divisions included UniProt, RefSeq, GenBank, "all other sequence databases" and sequences found in interaction records.

### Source data for interactions

Original molecular interaction data was downloaded from nine interaction databases detailed in Table 1. Interaction databases included BIND [22,23], BioGRID [24], DIP [25], HPRD [26,27], IntAct [28,29], MINT [30], MPact [31], MPPI [32] and OPHID [33]. Interaction records formatted in version 2.5 of the PSI-MI standard were used wherever possible. In the case of the BIND database, we experienced problems with the available PSI-MI 1.0 dataset and so BIND's flat files (tab-delimited text files) were used instead. These flat files are derived by parsing BIND database records written in the BIND data format [55]. BIND records that describe protein-protein interactions using EntrezGene identifiers are not properly included in this flat file and led to a high number of unassigned interactors (Table 3, column 4). Flat files and BIND-XML files are available from the authors upon request since they are no longer available from the BIND site now administered by Thomson Scientific as part of the BOND database [56,57]. OPHID is no longer updated and is being replaced by I2D [58].

### Parsing interaction data

PSI-MI XML files were processed using a Java parser employing the Streaming API for XML library (StAX) [59]. StAX is a pull-parser and does not generate a memory tree during operation; this allowed for processing of very large files without exceeding available RAM. Data retrieved from each interaction record included the interaction database name and record accession. Interaction record accessions were not provided by HPRD, MPPI or by OPHID.

Separate configuration files were written for each data source allowing the parser to handle both PSI-MI version 1.0 and 2.5 formatted files and variations in the use of the data structure by different interaction data providers. Interaction records providing evidence that some interaction does *not *occur were excluded from the consolidated interaction database (PSI-MI 2.5 Path: entrySet/entry/interactionList/interaction/negative) [60].

Parsing 3220 IntAct files (835 Mb) required 47 minutes using the hardware configuration described below. This time included reading XML files from disk, parsing and writing information back to the relational database.

### ROG assignments

Redundant object group (ROG) assignments were made for protein interactors as described in Figure 2 and the accompanying text in the results section. Assignment of approximately 628 thousand protein references to ROG's required 3 hours using the hardware configuration described below. This time included reading data from a relational database, making the assignment and writing information back to the relational database.

### Physical implementation for dataset production

All development work was performed on a single Linux workstation (2 X Intel(R) Xeon(TM) dual core 3.00 GHz CPUs each with 2 GB RAM). However, the backend relational database management processing required a more capable server-grade system, due to memory and disk storage space requirements. EMBNet Norway [61] provided a server with 2 quad core Xeon x86_64 processors and a total of 16 Gigabytes of RAM that ran a suitably tuned MySQL 5.0 database server [49].

The production of the datasets relied on the InnoDB storage engine [62] in order to ensure 'ACID' based transaction safety [63]. The MySQL InnoDB buffer was scaled to 8 Gigabytes (approximately 50% of the system RAM) ensuring that a large portion of table index operations were performed in memory and thus minimizing disk I/O operations as much as possible.

Additional server-side optimizations included the configuration of certain parameters of the underlying ext3 file system [64]. Briefly, a 4 Kbyte ext3 file system block size achieved fast file system response for MySQL and other backend processes creating large (several Gigabytes) files on average and a maximum file size of 2 Tbytes, essential to accommodate the creation of very large files for MySQL tables.

Finally, Ethernet link aggregation technology [65] was employed to connect the RDBMS server to a number of development workstations and central file storage areas, in order to increase the speed of secondary post production file access operations via the Network File System [66].

All these optimizations reduced the production of a typical dataset from weeks (non-optimized system) to a few days and produced a backend platform that could scale appropriately, as the size and number of integrated databases grows.

### Testing

#### PSI-MI validation and tag counts

We attempted to validate each of the PSI-MI input files against their relevant schemas (2.5 or 1.0). Only IntAct, MINT and HPRD files validated; all other files returned errors of various types including missing elements, identifiers and unexpected data types. This required that the PSI-MI file parser be customized for each input file in order to account for varying uses of the PSI-MI schema. We used an independent method to ensure our parser had found all interactors and interactions in input files. Tags indicating end of interactor and interaction elements were counted using text parsing methods. These counts exactly matched the number of elements retrieved using the XML parser on each of the input files. A similar analysis confirmed the number of protein interactors and interactions returned by our BIND flat-file parser.

Extensive spot-checking was performed during development and after the final build. In addition, we used web-services provided by IntAct and MINT to confirm the number of interactions and interactors parsed from those sources. No errors were found. Some interactions were returned that were not present in our data set because they were not included in the IntAct release that we parsed or because of differences between identifiers distributed in the XML file versus the web service.

### Lexicographical ordering of SEGUID's and ROGID's

SEGUID's are SHA-1 keys written in canonical base64 form [67] with trailing = characters removed. ROG identifiers concatenate a SEGUID with a numerical taxonomy identifier. Therefore, the allowable characters in a SEGUID or ROG identifier are (in ascending ASCII or Unicode value):

+/0123456789ABCDEFGHIJKLMNOPQRSTUVWXYZabcdefghijklmnopqrstuvwxyz

Lists of SEGUID or ROG identifiers were sorted in ascending ASCII-based lexicographical order. The comparison of two strings in a list is achieved by comparing the successive characters in each character index (starting from 0) until one string is determined to be greater than the other. The ASCII or Unicode value of each character determines if one character is 'less' than, 'equal' to or 'greater' than the other. This ordering was implemented using the Arrays.sort method in Java. The Perl sort function can be used to achieve equivalent results. Example code is provided with a test case on the iRefIndex FTP site (see below).

### Creation of RIGID's

A RIG identifier (RIGID) is constructed by concatenating ROG identifiers (after sorting them in ascending lexicographical order as described above), applying the SHA-1 algorithm to the resulting string, converting the digest to its base64 representation and removing all trailing "=" characters used for padding. It is important to note that the starting list includes a ROG identifier for *each *interactor listed in the interaction (even if these ROGID's are repeated). As a result, the RIGID takes into account the stoichiometry of the interactors present in an interaction; a complex composed of three A-type subunits will have a different RIGID than a complex with four A-type subunits.

## Availability and requirements

A subset of data consolidated by iRefIndex is available under a Creative Commons Attribution license [68] via anonymous FTP at  (user name: "ftp" password: "anonymous"). Presently, iRefIndex is updated manually by rebuilding the entire dataset. Releases will be accompanied by a detailed README file listing the release number, release date, a detailed description of the format and any change notices. No regular release schedule has been set at this time.

This index is provided in a tab-delimited, PSI-MITAB 2.5 text format [4]. Data in this format may be imported into the Cytoscape interaction viewer [69,70]; however, users are advised that importing the entire index into Cytoscape is likely to be time-consuming and they should instead first select those interactions (rows) of interest for visualization. Additional details are available at . iRefIndex provides a single entry for each distinct interaction group with links to source databases describing that interaction. ROG assignment scores are not provided but are available on request. Other distributions of iRefIndex data are possible and are actively being developed; this format was chosen in the hopes that it would prove immediately useful to the widest possible audience. Suggestions are welcome.

iRefIndex currently includes only those data sets that clearly can be redistributed under the licenses of the source databases. HPRD, DIP and MPACT are not included in the public distribution at this time. The entire index can be provided on request to academic researchers under a collaborative research agreement. Source databases interested in having their data included in the iRefIndex should contact the corresponding author. Source code is available on request under a GNU-GPL license.

## Authors' contributions

SR carried out all development work and participated in the system design. GM provided system's engineering support. IMD conceived of the study, participated in the system design and wrote the paper. All authors read and approved the final manuscript.
